# Environmental enrichment applied during epileptogenesis increases seizure latency and reduces serum interleukin-6 in a PTZ kindling model

**DOI:** 10.1007/s12031-026-02567-0

**Published:** 2026-07-21

**Authors:** Ana Carolina Sulzbach, Gabriel de Lima Rosa, Laura Gentilin Grotto, Rafael Bremm Padilha, Amanda Muliterno Domingues Lourenço de Lima, Edson Fernando Muller Guzzo, Brayan Braz Barbosa, Tainá Schons, Rosa Maria Martins de Almeida, Adriana Simon Coitinho

**Affiliations:** 1 Postgraduate Program in Biological Sciences, Pharmacology, and Therapeutics - Institute of Basic Health Sciences, Universidade Federal do Rio Grande do Sul, Rua Ramiro Barcelos, Porto Alegre, RS 2600 Brazil; 2 Postgraduate Program in Biological Sciences, Physiology - Institute of Basic Health Sciences, Universidade Federal do Rio Grande do Sul, Rua Ramiro Barcelos, Porto Alegre, RS 2600 Brazil; 3 Department of Microbiology, Immunology, and Parasitology - Institute of Basic Health Sciences, Universidade Federal do Rio Grande do Sul, Rua Ramiro Barcelos, Porto Alegre, RS 2600 Brazil; 4 Postgraduate Program in Biological Sciences, Biochemistry - Institute of Basic Health Sciences, Universidade Federal do Rio Grande do Sul, Rua Ramiro Barcelos, Porto Alegre, RS 2600 Brazil; 5 Postgraduate Program in Neurosciences - Institute of Basic Health Sciences, Universidade Federal do Rio Grande do Sul, Rua Ramiro Barcelos, Porto Alegre, RS 2600 Brazil; 6 Institute of Psychology, Laboratory of Experimental Psychology of Neuroscience and Behavior (LPNeC), Universidade Federal do Rio Grande do Sul, Porto Alegre, RS Brazil; 7 Departamento de Microbiologia, Imunologia e Parasitologia - ICBS, Universidade Federal do Rio Grande do Sul, Porto Alegre, Brazil Rua Ramiro Barcelos 2600 , RS 90035-003

**Keywords:** Epilepsy, Seizures, Environmental enrichment, Kindling, Animal model

## Abstract

Epilepsy is a chronic neurological disorder affecting millions of individuals worldwide and is strongly associated with inflammatory processes. Although pharmacological treatment remains the first-line approach, approximately one-third of patients are refractory to available therapies. Environmental enrichment (EE) has been shown to improve cognitive function, reduce stress, and enhance neuroplasticity, suggesting its potential as a non-pharmacological strategy for neurological disorders.

In this study, we investigated the effects of EE in a pentylenetetrazole (PTZ)-induced seizure model using different exposure paradigms. Animals were exposed either to EE for 30 days prior to seizure induction or during the 14-day PTZ kindling protocol. Seizure severity and latency were assessed, along with inflammatory (interleukin-6) and stress-related (cortisol) biomarkers.

EE exposure during seizure induction significantly increased the latency to seizure onset compared to both the control and pre-exposed groups, with a significant Group × Session interaction across the kindling protocol and pronounced effects at later PTZ sessions. A borderline trend toward reduced seizure severity was also observed in this group, but day-specific differences did not survive correction for multiple comparisons. The EED group also exhibited significantly lower serum IL-6 levels compared with controls. No significant differences in cortisol levels were observed in serum or in the frontal cortex. Pre-exposure to EE did not produce significant protective effects on any of the outcomes evaluated.

These findings suggest that EE exerts beneficial effects when applied during epileptogenic processes, particularly on the temporal progression of seizure susceptibility, and may be associated with changes in selected inflammatory markers. EE may represent a promising adjunct therapeutic strategy for epilepsy, although mechanistic studies with broader molecular characterization are still needed.

## Introduction

Epilepsy is a chronic neurological condition characterized by spontaneous and recurrent seizures. According to the World Health Organization (WHO), it affects 50 million people worldwide, with higher prevalence among children and the elderly (Beghi 2020). It is a debilitating condition and impacts every aspect of someone’s life, including physically, socially, and even professionally (Ali 2018).

An important research target in epilepsy is inflammation and its modulating role in the condition, mainly in epileptogenesis and neuronal activity modulation. The inflammatory process has already been implicated as a significant contributor in neurodegenerative diseases, and studies have shown that it is also involved in the genesis and progression of the epileptic crises (Vezzani 2014). Changes in the levels of the pro-inflammatory cytokines IL-6, IL1 beta and TNF-α have been reported in experimental models of epilepsy (Guzzo et al. 2018, 2024; de Lima Rosa et al. 2021).

Furthermore, elevated levels of the hormone cortisol have been reported in individuals with epilepsy, and this could be directly related to an increase in acute stress that is triggered by epileptic crises (Cano-López and González-Bono 2019).

Beyond inflammation and stress, oxidative stress also plays a central role in the pathophysiology of seizures and epileptogenesis. Excessive production of reactive oxygen species (ROS), particularly via NADPH oxidase (NOX) activation, contributes to neuronal hyperexcitability and damage in PTZ-induced seizure models (Singh et al. 2025). Antioxidant regulators such as nuclear factor erythroid 2-related factor 2 (Nrf2) (Carmona-Aparicio et al. 2015) and thioredoxin (Yu et al. 2019) counteract these effects by orchestrating the cellular antioxidant response and modulating neuroinflammation. Notably, combination antioxidant therapy has been shown to prevent epileptogenesis and modify the course of chronic epilepsy in experimental models (Shekh-Ahmad et al. 2019). The interplay between oxidative stress, neuroinflammation, and seizure susceptibility represents a promising mechanistic framework for understanding both epileptogenesis and the potential benefits of non-pharmacological interventions.

Being one of the most common neurological conditions, epilepsy has an established treatment that starts with medication therapy (Devinsky et al. 2018). Antiepileptic drugs (AED) or anti-seizure drugs (ASD) are the basis of epilepsy management, but although effective in most cases, studies revealed that around 30% of epilepsy patients have refractory episodes and do not benefit from the AEDs (Laxer et al. 2014; Shneker and Fountain 2003). For that, new alternatives, especially non-pharmacological ones, need to be studied and discovered.

Environmental Enrichment (EE) is a relatively recent term that has gained strength over the past few years. First mentioned by Donald O. Hebb, who detailed in his studies that rats who were raised as pets at home would perform better in cognitive tests rather than the ones kept in laboratory facilities (Brown and Milner 2003). EE is a practice whose objective revolves around improving animal housing conditions, including stimuli that make the environment more complex at cognitive, social, motor, and sensory levels (Rosenzweig et al. 1978). EE can be used in a variety of ways, and the protocols are always diverse and dependent on animal species and necessities (Kempermann 2019), but for laboratory animals, such as rats, it involves bigger or multiple cages, collective housing of animals on larger numbers, and the presence of stimuli such as tunnels, chewing toys, sheltering options or running wheels (Suemaru et al. 2018; Zeraati et al. 2021). It is of great interest to provide the animal with materials, toys, structures, or challenges that facilitate and stimulate the behaviors and characteristics typical of its species (Ratuski and Weary 2022).

Since Hebb’s studies, EE has collected a wide range of results demonstrating positive effects in various situations. It has already been demonstrated that EE can attenuate stress and anxiety symptoms and benefit animals cognitively, on learning and memory processes, also being capable of reducing aggressive and stress-related behaviors (Akyuz and Eroglu 2021; Macartney et al. 2022; Simpson and Kelly 2011). There are some investigations on the effects of EE in epilepsy (Akyuz and Eroglu 2021), but especially in a PTZ-induced epileptic seizure model (Dolu et al. 2013; Keloglan et al. 2016), these studies are scarce. Therefore, new studies need to be done. On this front, we investigated the effects of EE before and during an epileptic seizure animal model, with the hypothesis of EE having protective effects on the seizures.

## Materials and Methods

### Animals

Male Wistar rats, 1 month old, weighing approximately 100 g, from the Center for Reproduction and Experimentation of Laboratory Animals (CREAL) at Universidade Federal do Rio Grande do Sul (UFRGS) were used in the study. The animals had free access to water and food and were kept in polypropylene boxes measuring 41 cm x 34 cm x 16 cm, with a maximum of 4 animals per box, in an environment with a 12-hour light-dark cycle (07:00 AM − 07:00 PM) at a temperature of 23 ± 1 °C. The animals were kept at the CREAL. The experiments were approved by the ethical committee of the Universidade Federal do Rio Grande do Sul and the Ethical Committee of Animal Usage (CEUA), under project number 44,316, and followed the ARRIVE guidelines.

### Experimental Groups

The animals were randomly divided into three groups (*n* = 10–12): one group that was exposed to EE for 30 days exclusively before applying the kindling model (EEB); one group that was exposed to EE only during the 14 days of the kindling model (EED) and, lastly, a control group (C) that remained without any type of enrichment, both before and during the application of the kindling model.

### Environmental Enrichment

**Fig. 1:**
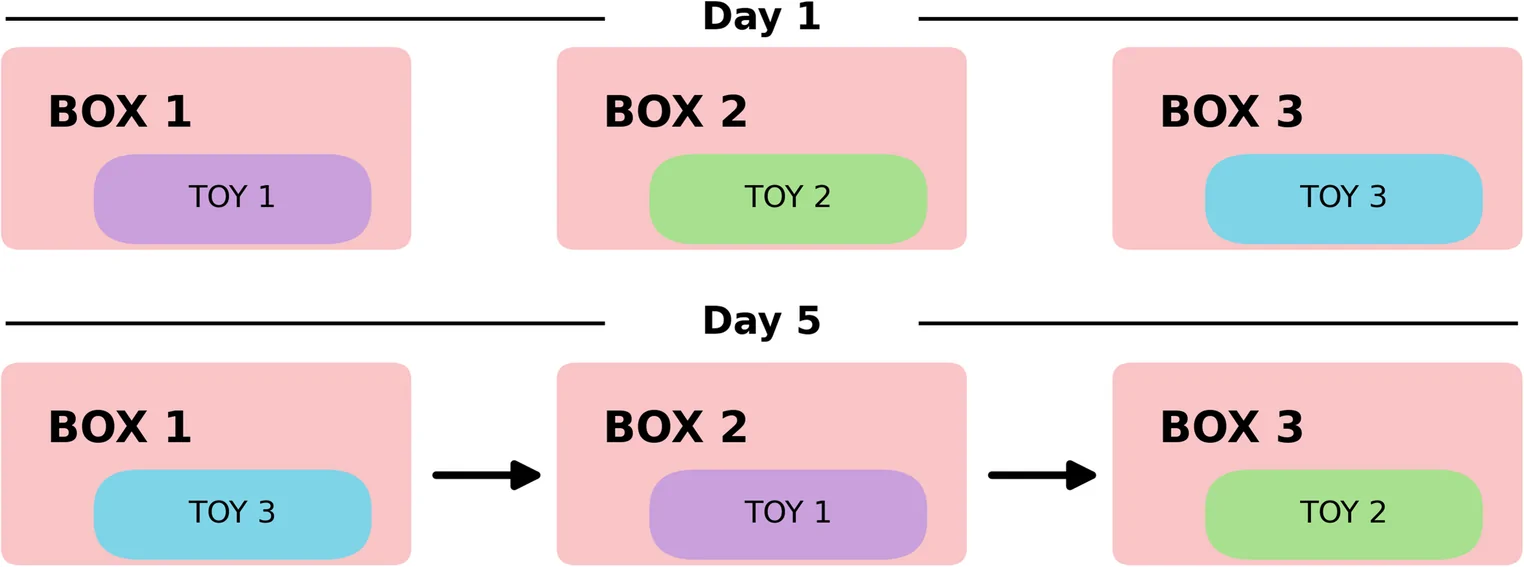
Demonstration of the distribution and alternation of the wooden and calcium toys. The toys were alternated in 4 to 5-day intervals, following a pattern for each box to experience every kind of toy at least once

The groups of enriched animals were housed in boxes with high bars, which increased the proportions of the boxes to 41 cm x 34 cm x 24 cm. Furthermore, the boxes contained a hanging tricoline hammock measuring 25 cm x 25 cm and various wooden and calcium toys, as well as materials such as toilet paper rolls and pieces of paper, which were used as improvised toys (tunnels and food-based toys). The knitting nets were present in all the boxes and were maintained every day, and the improvised toys were remade every day, always being available. The various wooden and calcium toys were rotated between the boxes of enriched animals at intervals of 4 to 5 days (Fig. 1). Lastly, the control group was housed in common boxes without any enrichment.

### Behavioural Activity and Kindling

**Fig. 2:**
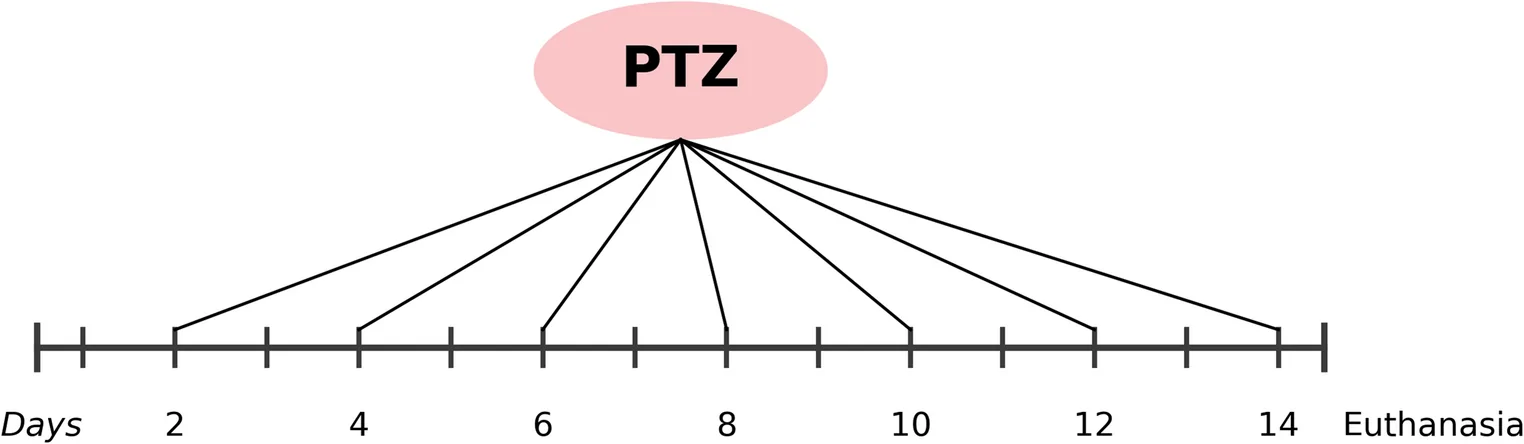
Demonstrative figure of the PTZ administration pattern. PTZ was administered on alternate days for 14 days with a total of 7 administrations. On the 15th day, the euthanasia was conducted

**Fig. 3:**
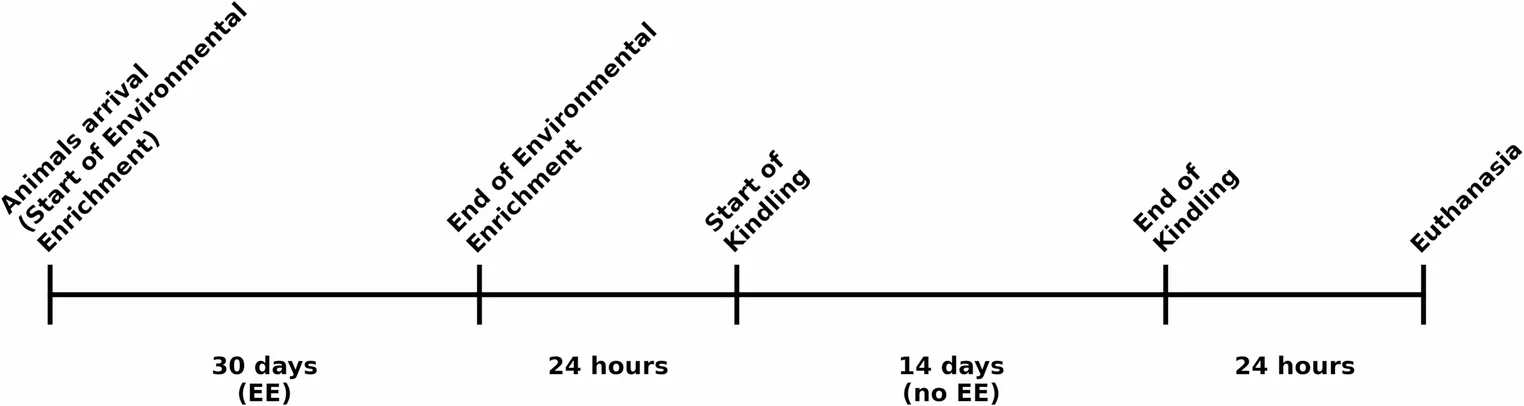
Experiment timeline for the EEB group (environmental enrichment before kindling). Animals received 30 days of environmental enrichment immediately after arrival, followed by a 24-hour interval before the 14-day PTZ kindling protocol, during which no enrichment was provided. Euthanasia was performed 24 h after the last PTZ administration

**Fig. 4:**
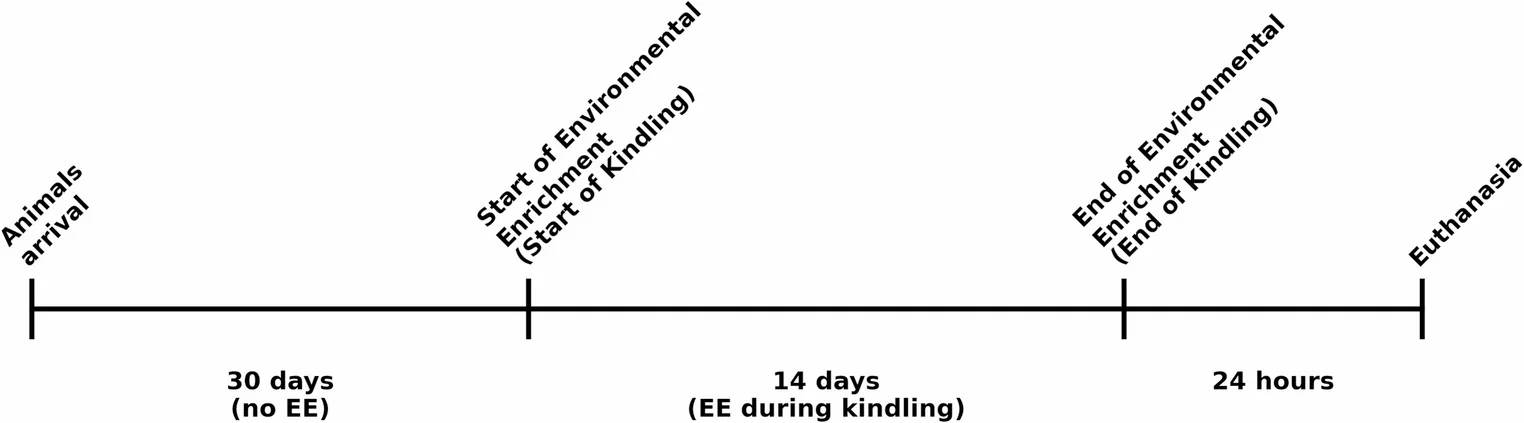
Experiment timeline for the EED group (environmental enrichment during kindling). Animals were maintained under standard housing for 30 days after arrival; environmental enrichment was then introduced and maintained throughout the 14-day PTZ kindling protocol. Euthanasia was performed 24 h after the last PTZ administration

From the thirty-second day of experimentation, on alternate days over a period of 14 days, the kindling model of seizures was used. In this protocol, the animals received sub-convulsant doses (30 mg/kg) intraperitoneally of the pro-convulsant drug pentylenetetrazole (PTZ) on alternate days, totaling seven administrations (Figs. 2 and 3, and 4). After PTZ administration, the animals underwent 20 min of observation in which the severity of the seizures was graded according to the Racine scale (Racine 1972). The latency to the onset of the first epileptic seizure was also registered. Animals that did not develop seizures within the 20-minute observation window were assigned the maximum observation time value (1200 s) for latency analyses. All behavioral assessments (Racine severity scoring and latency measurements) were performed by an experimenter blinded to group allocation throughout the entire kindling protocol. 

### Brain Microdissection and Tissue Preparation

On the 15th day, the animals were sacrificed by decapitation, and the brain tissue was immediately removed and kept on an ice-plate for dissection. The frontal cortex and hippocampus, from both hemispheres, were dissected and homogenized with 1:10 (mass/volume) PBS buffer. The homogenate was centrifuged at 800 g for 10 min, and the supernatant was collected for biochemical and immunological assays. For serum, blood from the brainstem was collected and centrifuged at 1000 g for 5 min. All samples were stored at − 80 °C.

### Immunological Assays

Interleukin-6 (IL-6) and cortisol levels were quantified using commercially available enzyme-linked immunosorbent assay (ELISA) kits (BT LAB, Jiaxing, China), according to the manufacturer’s instructions. Samples from serum, hippocampus, and frontal cortex were analyzed in duplicate. Absorbance was measured at 450 nm using a microplate reader (Biochrom Anthos Zenyth 200rt), and concentrations were determined based on standard curves. Results were expressed as pg/mL for serum and pg/mg protein for brain tissues. Biochemical analyses were performed in a randomly selected subset of animals (*n* = 5 for serum and *n* = 4 for tissues per group), selected prior to unblinding of group allocation. Tissue IL-6 (hippocampus and frontal cortex) and cortisol (serum and frontal cortex) were assessed in the control and EED groups, owing to sample availability.

### Statistical Analysis

Longitudinal behavioral outcomes (seizure severity scores and latency to seizure onset) were analyzed using repeated-measures analysis of variance (ANOVA), with group (C, EEB, EED) as the between-subjects factor and PTZ session (1–7) as the within-subjects factor. Latency values were log-transformed prior to analysis to approximate normality. Mauchly’s test was used to assess sphericity, and the Greenhouse–Geisser correction was applied to the within-subjects effects when this assumption was violated. Box’s M test was used to verify the homogeneity of covariance matrices across groups. When significant main effects or interactions were detected, pairwise comparisons of estimated marginal means were performed with Bonferroni correction to control the family-wise error rate.

Biochemical outcomes (IL-6 and cortisol) measured at the experimental endpoint were analyzed after verifying normality with the Shapiro–Wilk test and homogeneity of variances with Levene’s test. When assumptions were satisfied, parametric tests were used (one-way ANOVA followed by Tukey’s post hoc for three-group comparisons; Student’s t-test for two-group comparisons). Non-parametric alternatives (Kruskal–Wallis followed by Dunn–Bonferroni, or Mann–Whitney) were applied otherwise. Data are presented as mean ± standard deviation (SD) for parametric variables and as median and interquartile range (IQR) for non-parametric variables. A p-value < 0.05 was considered statistically significant. All statistical analyses were performed using SPSS software (version 18.0).

## Results

### Effect of Environmental Enrichment on Latency to Seizure Onset

Mauchly’s test indicated that the sphericity assumption was met for log-transformed latency (W = 0.39, *p* = 0.163). The repeated-measures ANOVA revealed a significant main effect of PTZ session (F(6, 180) = 24.72, *p* < 0.001, partial η² = 0.452), consistent with progressive kindling across sessions, a significant main effect of Group (F(2, 30) = 6.31, *p* = 0.005, partial η² = 0.296), and a significant Group × Session interaction (F(12, 180) = 3.07, *p* < 0.001, partial η² = 0.170).

Bonferroni-corrected pairwise comparisons of the main effect of Group indicated that the EED group exhibited significantly longer latencies than both the control group (mean difference = 0.51 log-units, *p* = 0.021) and the EEB group (mean difference = 0.55 log-units, *p* = 0.009). The control and EEB groups did not differ from each other (*p* = 1.000), indicating that pre-kindling enrichment alone, without continued exposure during seizure induction, was not sufficient to produce a measurable protective effect on latency.

**Fig. 5:**
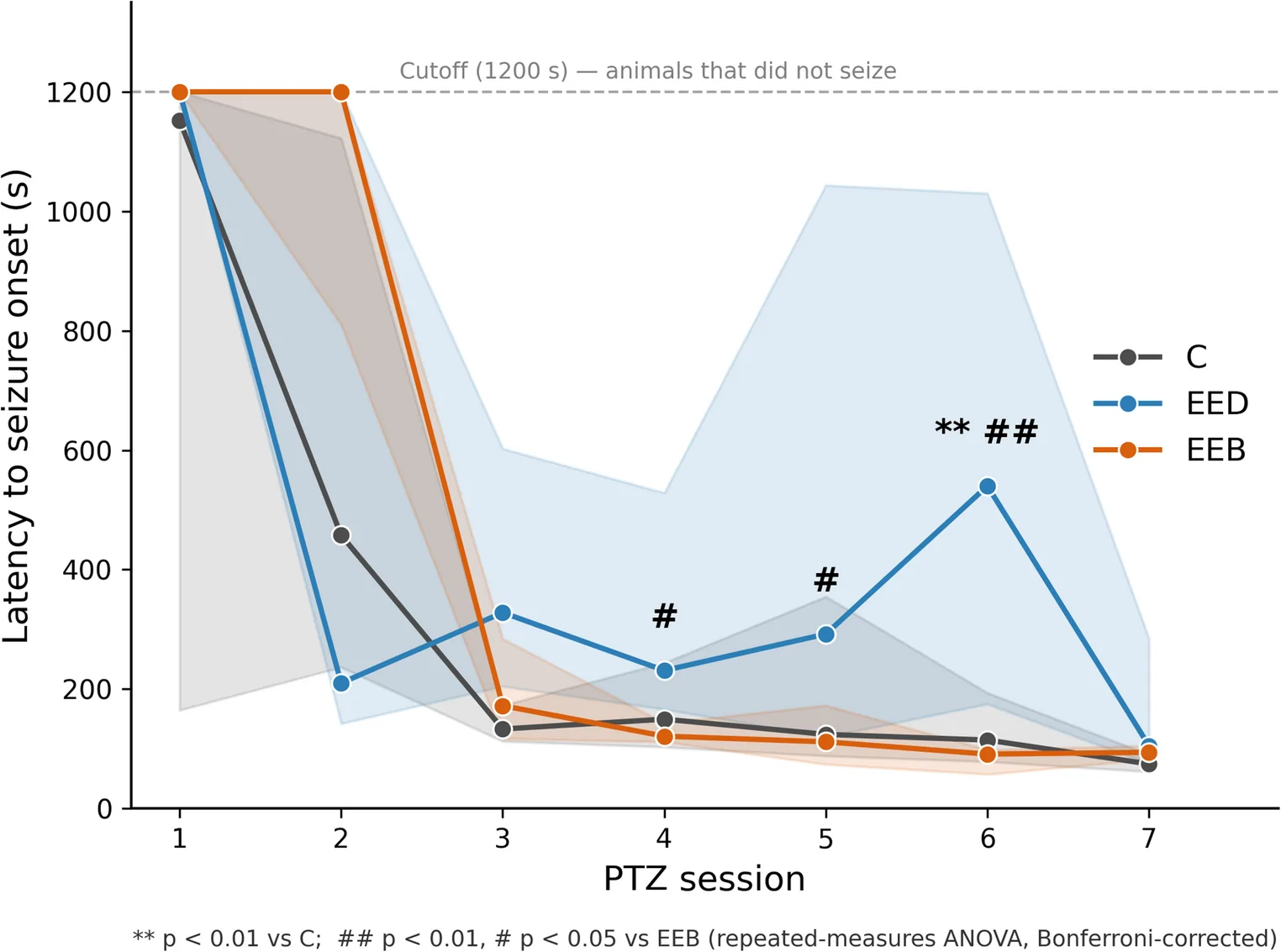
Effect of environmental enrichment on the latency to seizure onset across the seven PTZ sessions. Results are expressed as median (line) and interquartile range (shaded area). Analysis was performed using repeated-measures ANOVA on log-transformed latency values, with Bonferroni-corrected post-hoc pairwise comparisons. ** *p* < 0.01 vs. control (C) group; ## *p* < 0.01, # *p* < 0.05 vs. EEB group. The x-axis represents the seven PTZ administrations (sessions 1–7). *n* = 10–12 animals per group. EEB: Environmental enrichment before the kindling model. EED: Environmental enrichment during the kindling model

Bonferroni-corrected pairwise comparisons within each PTZ session further showed that the EED group displayed significantly longer latencies than the EEB group at sessions 4 (*p* = 0.047), 5 (*p* = 0.036), and 6 (*p* < 0.001), and significantly longer latencies than the control group at session 6 (*p* = 0.005). The protective effect of EED was particularly pronounced at session 6, where the simple effect of Group accounted for nearly half of the between-subjects variance (F(2, 30) = 14.91, *p* < 0.001, partial η² = 0.499) (Fig. 5).

To verify the robustness of the latency findings to the assignment of the maximum observation time (1200 s) to animals that did not seize, a sensitivity analysis using a linear mixed-effects model restricted to the subset of uncensored observations (*n* = 181 of 231; 78.4%) was performed. This analysis confirmed the significant main effect of PTZ session and yielded a borderline Group × Session interaction (*p* = 0.07), consistent with the main analysis when the reduced sample size is taken into account.

### Effect of Environmental Enrichment on Seizure Severity

**Fig. 6:**
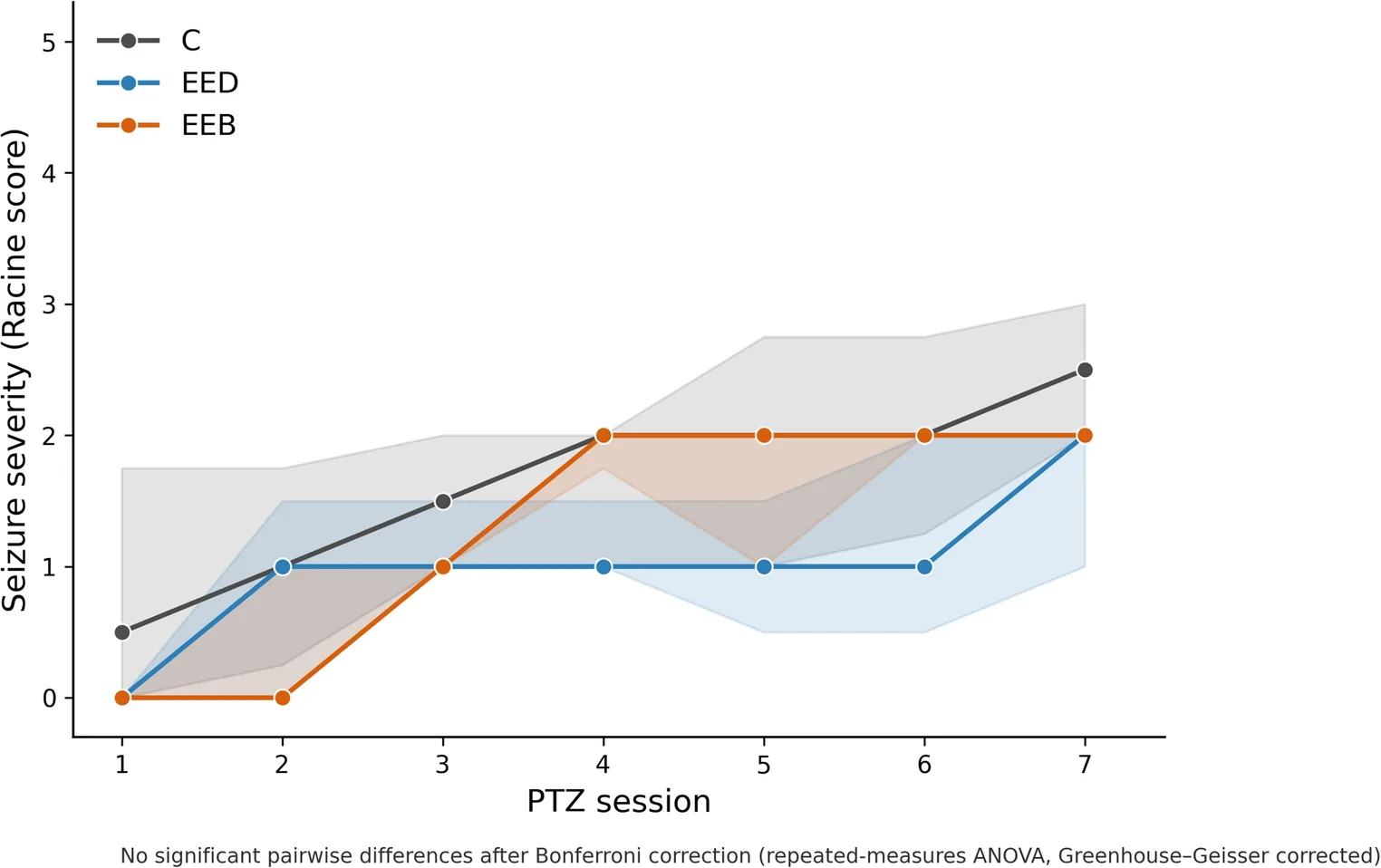
Effect of environmental enrichment on seizure severity (Racine score) across the seven PTZ sessions. Results are expressed as median (line) and interquartile range (shaded area). Analysis was performed using repeated-measures ANOVA with Greenhouse–Geisser correction and Bonferroni-corrected post-hoc pairwise comparisons. No pairwise comparison reached significance after correction for multiple comparisons. The x-axis represents the seven PTZ administrations (sessions 1–7). *n* = 10–12 animals per group. EEB: Environmental enrichment before the kindling model. EED: Environmental enrichment during the kindling model

For seizure severity (Racine scores), Mauchly’s test indicated a violation of sphericity (W = 0.28, *p* = 0.019), and Greenhouse–Geisser-corrected statistics were therefore used. The repeated-measures ANOVA revealed a significant main effect of PTZ session (F(4.14, 124.34) = 26.39, *p* < 0.001, partial η² = 0.468), consistent with progressive kindling across sessions, a borderline main effect of Group (F(2, 30) = 2.79, *p* = 0.077, partial η² = 0.157), and no significant Group × Session interaction (F(8.29, 124.34) = 1.14, *p* = 0.340, partial η² = 0.071). No pairwise comparison reached significance after Bonferroni correction, although the difference between the control and EED groups approached significance (mean difference = 0.57, *p* = 0.082) (Fig. 6).

### Effect of Environmental Enrichment on Cortisol Levels

**Fig. 7:**
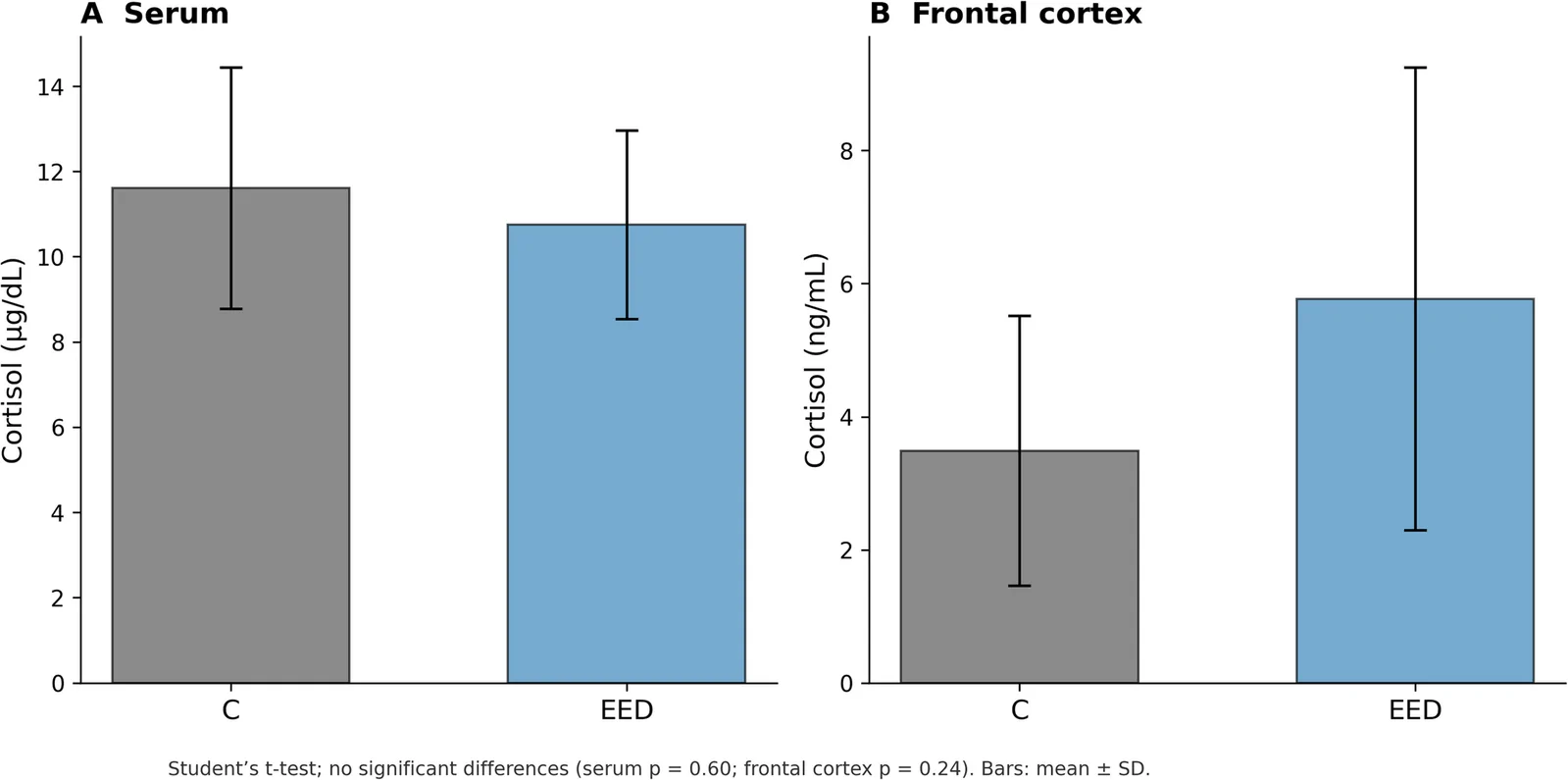
Effect of EE on cortisol levels in serum (Panel **A**) and frontal cortex (Panel **B**), comparing the control and EED groups. Results are expressed as mean ± SD. Student’s t-test; no significant differences were observed between groups (serum: control *n* = 6, EED *n* = 5, *p* = 0.60; frontal cortex: control *n* = 5, EED *n* = 5, *p* = 0.24). EED: Environmental enrichment during the kindling model

Cortisol levels were measured in serum and frontal cortex of the control and EED groups. No significant differences were observed between groups in serum cortisol (control: 11.61 ± 2.83 µg/dL, *n* = 6; EED: 10.75 ± 2.21 µg/dL, *n* = 5; Student’s t-test, t(9) = 0.55, *p* = 0.60) or in frontal cortex cortisol (control: 3.49 ± 2.03 ng/mL, *n* = 5; EED: 5.77 ± 3.48 ng/mL, *n* = 5; Student’s t-test, t(8) = -1.27, *p* = 0.24) (Fig. 7).

### Effect of Environmental Enrichment on IL-6 Levels

**Fig. 8:**
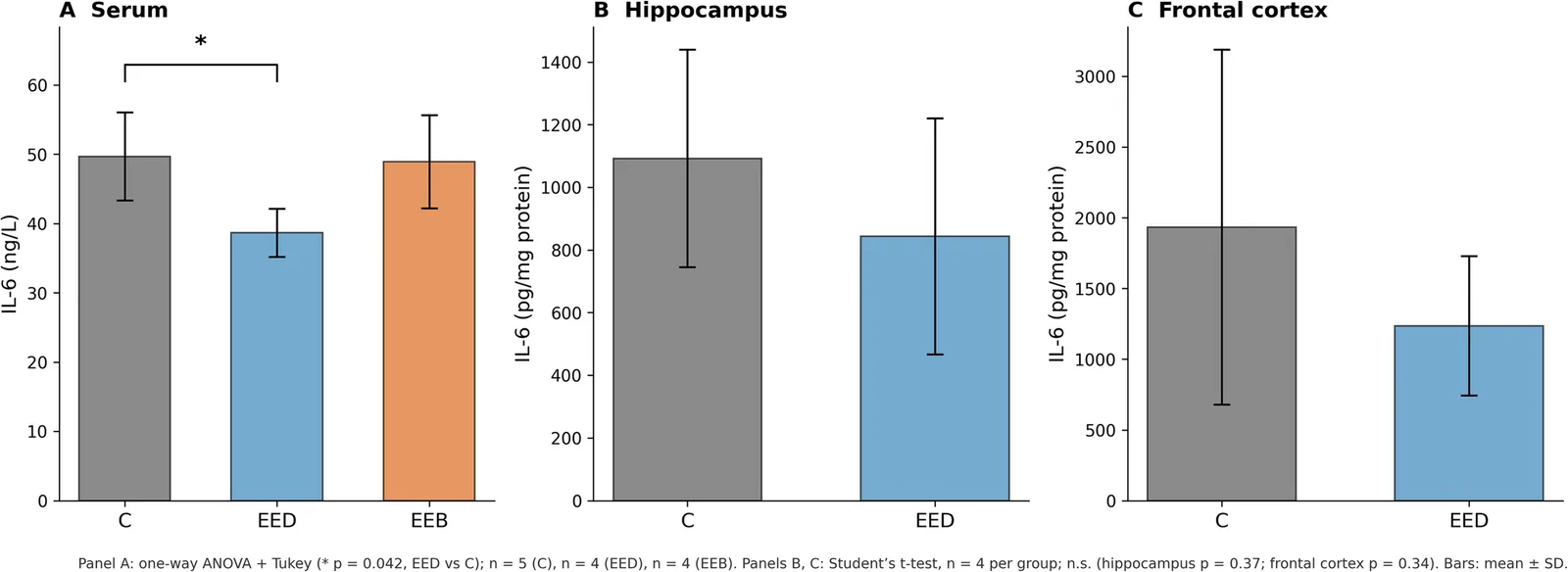
Effect of EE on IL-6 levels in serum (Panel **A**), hippocampus (Panel **B**), and frontal cortex (Panel **C**). Panel A compares the three groups using one-way ANOVA followed by Tukey’s post hoc test (C: *n* = 5; EED: *n* = 4; EEB: *n* = 4); * *p* = 0.042 vs. control group. Panels B and C compare the control and EED groups (Student’s t-test; *n* = 4 per group); no significant differences between groups in either tissue (hippocampus *p* = 0.37; frontal cortex *p* = 0.34). Results are expressed as mean ± SD. EEB: Environmental enrichment before the kindling model. EED: Environmental enrichment during the kindling model

Serum IL-6 levels were significantly reduced in the EED group compared with the control group, as revealed by one-way ANOVA followed by Tukey’s post hoc test (C: *n* = 5; EED: *n* = 4; EEB: *n* = 4; F(2, 10) = 4.76, *p* = 0.035; Tukey C vs. EED *p* = 0.042; Tukey C vs. EEB *p* = 0.98; Tukey EED vs. EEB *p* = 0.073). Tissue IL-6 measurements (frontal cortex and hippocampus; *n* = 4 per group) were performed in the control and EED groups. Normality was confirmed by Shapiro–Wilk test (frontal cortex: p_C = 0.39, p_EED = 0.23; hippocampus: p_C = 0.21, p_EED = 0.83) and variance homogeneity by Levene’s test (frontal cortex: *p* = 0.19; hippocampus: *p* = 0.79). Student’s t-test revealed no significant differences between groups in IL-6 levels in either tissue (frontal cortex: t(6) = 1.04, *p* = 0.34; hippocampus: t(6) = 0.97, *p* = 0.37) (Fig. 8).

## Discussion

Epilepsy is a very debilitating condition, and despite all the pharmacological treatments, up to a third of the patients will still experience refractory episodes. The existing non-pharmacological alternatives, such as surgery, are expensive and, besides being considered only for a few cases, are also commonly not available to a lot of the patients who live in developing countries, where epilepsy is more predominant (Fiest et al. 2017). This shows the need to look further into other simpler and possibly cheaper treatment alternatives, such as EE.

Evidence suggests that EE is highly effective in mitigating stress levels, enhancing cognitive functions such as memory and learning, and protecting against neurodegenerative conditions. Each of these outcomes has been seen in epilepsy researches as positive effects of EE demonstrated a significant correlation with epilepsy in various studies (Dandi et al. 2023; Macartney et al. 2022; Smail et al. 2020). The results obtained in this investigation supported the benefits already observed by these other investigations, which were able to find a protective effect that is very likely to have originated from EE exposure. EE does affect the brain in epilepsy-related pathways, such as hippocampal neurotrophic functions, which are increased by EE, benefiting neuronal cells in growth, development, and plasticity (Angelucci et al. 2009; Cao et al. 2014; Ickes et al. 2000).

Additionally, while researchers were able to find similar results regarding a protective effect promoted by EE, there are not many observations relating to EE exposure in animal models of PTZ-induced epileptic seizures. In this study, we verified the effects of EE done both before (EEB group) and during (EED group) the application of the PTZ-induced kindling model. Therefore, the animals were exposed to EE at different moments, for different periods of time. The revised statistical analysis revealed a robust protective effect of EED on latency to seizure onset, with a significant Group × Session interaction and Bonferroni-corrected pairwise differences emerging at multiple PTZ sessions. By contrast, the previously reported day-specific differences in seizure severity did not survive correction for multiple comparisons. While the overall direction of the severity findings (with the EED group tending to show lower scores) is consistent with a protective influence, the present data support a more cautious interpretation, in which the most robust effect of EED is on the temporal progression of seizure susceptibility as reflected by latency to seizure onset, rather than on the severity of individual seizure episodes. Importantly, the experimental design does not allow a strict distinction between preventive and therapeutic effects of EE: the EEB group underwent enrichment only before PTZ administration, whereas the EED group remained exposed to enrichment during kindling. The observed differences may therefore reflect both the timing and the persistence of the intervention. The finding that the control and EEB groups did not differ from each other (*p* = 1.000 for the latency main effect comparison) suggests that pre-kindling enrichment alone, in the absence of continued exposure, was not sufficient to produce a measurable protective effect — supporting an interpretation centered on the role of sustained enrichment exposure during the epileptogenic process, rather than on enrichment timing per se. This effect could be related to inflammatory pathways, which have been found to be connected to epilepsy (de Lima Rosa et al. 2021; Vezzani 2014). Epilepsy causes brain inflammation (Rana and Musto 2018), and exposure to EE during seizure induction could ameliorate the inflammatory response caused by the seizures. We were able to test this idea and, through the result of lower serum IL-6 levels in the EED group, observed it as consistent with this hypothesis. A 2020 study (Scarola and Bardi 2021) on inflamm-aging reported that IL-6 levels in enriched animals did not significantly change with aging, but non-enriched animals had a significant IL-6 proliferation in their adulthood, demonstrating an interesting modulating capacity regarding EE. Beyond inflammation, oxidative stress represents an additional mechanistic pathway that may contribute to the protective effects of EE in PTZ-induced epileptogenesis. PTZ-induced seizures are associated with increased generation of reactive oxygen species (ROS) and activation of NADPH oxidase (NOX), which contribute to neuronal hyperexcitability and oxidative damage; recent work has shown that selective NOX2 inhibition mitigates seizure susceptibility and neuroinflammation in this very model (Singh et al. 2025). Antioxidant regulatory systems, notably Nrf2 (Carmona-Aparício et al. 2015) and thioredoxin signaling, counteract these effects by promoting the cellular antioxidant response and modulating neuroinflammation; thioredoxin-related antioxidant proteins are themselves up-regulated in the cortex of PTZ-kindled animals, supporting their endogenous role in this paradigm (Yu et al. 2019). Indeed, combination antioxidant therapy has been reported to prevent epileptogenesis and modify the course of chronic epilepsy in experimental models (Shekh-Ahmad et al. 2019), supporting the relevance of these pathways as therapeutic targets. Environmental enrichment has been described to influence both inflammatory and oxidative stress pathways, and the interactions among these pathways may underlie the integrated protective effect of EE observed in our experimental model. Although a direct evaluation of oxidative stress markers was beyond the scope of this initial study, this mechanistic framework provides a relevant background for the interpretation of our inflammatory findings and points to a clear direction for future investigations.

As already mentioned before, EE has shown effect regarding stress levels at a behavioral level but also at molecular levels. Studies in various animal species demonstrated that EE can lower cortisol levels (Casal et al. 2017; Wojtaś et al. 2024). In the present study, however, no significant differences in cortisol levels were observed between the EED and control groups in serum or frontal cortex. The use of cortisol rather than corticosterone, the predominant glucocorticoid in rodents, represents a limitation of this study, and the cortisol findings should be interpreted as exploratory. Future studies should incorporate corticosterone measurements to provide a more accurate characterization of the stress-related effects of EE in this model.

The study from Dolu and colleagues (Dolu et al. 2013), which used the PTZ kindling model, found that the enriched animals had lower stress levels and sympathetic activity. Another research that utilized the PTZ kindling model also showed that EE reversed impairments caused by the seizure induction (Keloglan et al. 2016). Both studies showed benefits and protective effects regarding EE, which are consistent with our latency results. In this study, though, we used a 14-day protocol for the kindling model; meanwhile, the previously mentioned investigations used protocols with more than 30 days.

Aside from the PTZ-induced kindling model, other epilepsy models have observed benefit from EE on the condition. Investigations on a status epilepticus model using lithium and pilocarpine found that enriched animals performed better on memory and learning tests than the animals in standard housing (Faverjon et al. 2002; Rutten et al. 2002; Wang et al. 2007). Also, a temporal lobe epilepsy kainate model submitted animals to an early EE exposure - right after kainic acid lesion - and late EE exposure − 60 days after kainic acid lesion - and found that both early and late exposures to EE had the animals performing better on behavioral tests (Gorantla et al. 2019).

Even though the previously mentioned results do support the results in this study, it is complex to actually compare them due to the lack of investigations with this protocol. As mentioned, there are a few studies that made use of the PTZ-induced epileptic seizures model, but both had a larger timeline on PTZ administration (Dolu et al. 2013; Keloglan et al. 2016). Thus, with time and more studies carried out, it will be possible to obtain better comparisons with the obtained results.

### Limitations

Several limitations of this study should be acknowledged. First, the EE protocols are highly variable across the literature (Kempermann 2019), and behavioral interaction with enrichment objects was not quantified, limiting the reproducibility of the paradigm. Standardized metrics of activity and object engagement would strengthen future studies. Second, only male animals were included, which limits the generalizability of the findings; sex-specific differences in seizure susceptibility, stress responses, and neuroinflammation warrant further investigation. Third, the biochemical analyses were performed in a relatively small subset of animals (*n* = 4–5 per group), reducing the statistical power of these comparisons and increasing the risk of type II errors. Biochemical analyses were also limited to a subset of groups and tissues, which restricts direct comparisons across all experimental conditions. Fourth, only IL-6 was measured as an inflammatory marker; future studies should include additional cytokines (IL-1β, TNF-α), glial activation markers (GFAP, Iba1), and neurotrophic factors (BDNF) to allow stronger mechanistic conclusions about neuroinflammation. Fifth, cortisol rather than corticosterone was used as a stress marker; given that corticosterone is the predominant rodent glucocorticoid, the cortisol findings should be interpreted with caution. Sixth, animals that did not develop seizures within the 20-minute observation window were assigned the maximum observation time value (1200 s) for latency analyses; a sensitivity analysis on uncensored observations confirmed the main findings, but the censoring procedure may have influenced effect size estimates, particularly for early PTZ sessions. Finally, the experimental design does not allow a strict distinction between effects driven by the timing of EE exposure and effects driven by its persistence during kindling, since the EEB and EED protocols differ in both dimensions. Behavioral assessments beyond seizure severity and latency (e.g., anxiety, cognition, locomotor activity) were also not included in the present design and would provide a broader characterization of the effects of EE in future studies. Notwithstanding these limitations, the blinded assessment of seizure severity and latency throughout the kindling protocol represents a methodological strength supporting the reliability of the behavioral findings.

## Conclusion

In this study, animals exposed to environmental enrichment during a PTZ-induced kindling protocol showed a robust and sustained increase in latency to seizure onset across the kindling progression, with significant effects emerging at later PTZ sessions, and a significant reduction in serum interleukin-6 levels. No significant differences were observed in seizure severity after correction for multiple comparisons, or in cortisol levels in serum or frontal cortex. Pre-exposure to EE before the kindling protocol did not produce significant effects on any of the outcomes evaluated. Taken together, these findings suggest that EE applied during the kindling protocol is associated with increased seizure latency and reduced serum IL-6. Broader claims regarding anti-inflammatory, antioxidant, or disease-modifying effects of EE would require additional direct measurements and are not made here. Further studies incorporating broader molecular characterization, corticosterone measurements, and behavioral assessments will be necessary to clarify the mechanisms underlying these effects. 

Figures are presented below, following the order of citation in the text. Figures 1, 2, 3 and 4 illustrate the enrichment protocol and experimental timeline; Figs. 5, 6, 7 and 8 present the experimental results. 

## Data Availability

The anonymized database utilized in this study can be obtained from the corresponding author upon reasonable request.
